# Supplementary material for: Content-rich biological network constructed by mining PubMed abstracts
Source: BMC Bioinformatics. 2004 Oct 8;5:147. doi: 10.1186/1471-2105-5-147 (PMC528731; doi:10.1186/1471-2105-5-147)
Supplement: Additional File 2 — The original results of the above study (non-essential files are deleted to keep the file size under the limit set by BMC bioinformatics). [file 1471-2105-5-147-S2.bz2 › chilibotAdditionalFile2/dip20/386ID10318834E9856/html/EPO_EPOR.html]

 


 **EPO** and **EPOR** 
  
Found 716 abstracts in PubMed, retrieved 20.  
 

 What does Google say? 
 PDF only 
| .edu only 

---

**Interactive relationship** (e.g. stimulation, inhibition, etc)

**Neutral relationship**- Both  **Epo**  and  **EpoR**  are expressed by astrocytes and neurons and  **Epo**  is present in the cerebrospinal fluid CSF.  Ref: 12638727 J Neuropathol Exp Neurol, 2003
- Cytoprotection by  **EPO**  is initiated by the activation of the  **EPO**  receptor  **EPOR**  and subsequent signal transduction pathways that originate with the Janus tyrosine kinase 2 Jak2 protein.  Ref: 12769640 Curr Drug Targets Cardiovasc Haematol Disord, 2003
- Erythropoietin  [ **EPO** ]   **EPO**  regulates mammalian erythropoiesis by binding to its transmembrane receptor  **EPOR** .  Ref: 12878211 Biochem Biophys Res Commun, 2003
- A characteristic cluster upregulated in the  **EPO**  R  [ **EPOR** ]  and  **EPO**  W O groups and downregulated in the  **EPO**  H group was identified that contained various cytokines and growth factors, including IL 6, GM CSF, vascular endothelial growth factor B, IL 9, IL 3, leukemia inhibitory factor, and interferon alpha 2, and such receptors as thrombopoietin receptor, IL 9 receptor, and colony stimulating factor 1 receptor.  Ref: 12612986 Am J Kidney Dis, 2003
- These findings indicate the expression of multiple  **EPOR**  isoforms in human cancer cells that may modulate the cellular effects of recombinant human  **EPO**  or  **EPO**   **EPOR**  antagonists.  Ref: 12878211 Biochem Biophys Res Commun, 2003
- The multifunctional role of  **Epo**  has further been confirmed by the discovery in the central nervous system of a specific  **Epo**   **Epo**  receptor  **EpoR**  system.  Ref: 12638727 J Neuropathol Exp Neurol, 2003
- Signals provided by the  **Epo**  receptor  **EpoR**  are required for erythroid development beyond the CFUe stage, and are propagated via the  **EpoR**  tethered Janus kinase, JAK2.  Ref: 12869513 BloodBlood, 2003
- Presently, the activities of a PY null  **EpoR**  HM form in primary progenitor cells from knock in mice were further assessed using optimized  **Epo**  dose dependent proliferation, survival and differentiation assays.  Ref: 12869513 BloodBlood, 2003
- Strong evidence indicates that erythropoietin  [ **EPO** ]   **Epo**  is eliminated via  **Epo**  receptors  **EpoR** .  Ref: 12750427 J Pharmacol Exp Ther< MedlineTA>J Pharmacol Exp Ther, 2003

**Non-interactive relationship** (e.g. studied together, co-existance, homology, etc.)

- Stage IV gastric carcinoma had a higher degree of vascularization than other stages, and  **Epo**  R  [ **EPOR** ]  expression in both endothelial and tumour cells increased in parallel with malignancy grade and was highly correlated with the extent of angiogenesis.  Ref: 12605644 Histopathology, 2003
- METHODS Reverse transcriptase polymerase chain reaction, immunohistology, and Western blots were used to establish the presence and localization of  **EPO**  R  [ **EPOR** ]  on rat, nonhuman primate, and human islets.  Ref: 12717230 Transplantation, 2003
- RESULTS The presence of  **EPO**  R  [ **EPOR** ]  was demonstrated on islets regardless of species.  Ref: 12717230 Transplantation, 2003
- Furthermore, administration of  **EPO**   **EPOR**  antagonists delayed the growth of uterine, ovarian, and mammary carcinoma cells in experimental animal models.  Ref: 12878211 Biochem Biophys Res Commun, 2003
- It has previously been demonstrated that mutations in the 232 WSAWS 236 motif in the erythropoietin  [ **EPO** ]  receptor  **EPOR**  can result in strongly inhibited surface expression, due to defective intracellular transport Hilton, D.  Ref: 12859190 Biochemistry, 2003
- CONCLUSIONS  **Epo**  R  [ **EPOR** ]  level correlates with angiogenesis and progression of patients with gastric carcinoma and we suggest that  **Epo**  might have a trophic effect on the vasculature of the gastrointestinal tract.  Ref: 12605644 Histopathology, 2003
- Using immunohistochemistry, we examined the expression of  **Epo** ,  **EpoR** , p16, hypoxia inducible factor HIF 1alpha, and bcl 2 in benign and dysplastic cervical squamous epithelia and invasive squamous cell carcinomas ISCCs.  Ref: 12759237 Am J Pathol, 2003
- CONCLUSIONS This is the first demonstration that pancreatic islets express  **EPO**  R  [ **EPOR** ] .  Ref: 12717230 Transplantation, 2003
- n = 10, rHuEPO responsive non HD patients with CRF  **EPO**  R  [ **EPOR** ] .  Ref: 12612986 Am J Kidney Dis, 2003
- As compared to the wt  **EpoR** ,  **EpoR**  HM activity was compromised several fold in each context when  **Epo**  was limited to physiological concentrations.  Ref: 12869513 BloodBlood, 2003
- In this study we correlated microvascular density and  **Epo**  receptor  **Epo**  R  [ **EPOR** ]  expression in endothelial and tumour cells with histopathological type in gastric cancer.  Ref: 12605644 Histopathology, 2003
- Assuming that  **Epo**  s elimination largely depends not only on erythropoietic  **EpoR** , but also non hematopoietic  **EpoR** , this work shows a significant difference in the relative proportions of the two  **EpoR**  populations in lamb and adult sheep.  Ref: 12750427 J Pharmacol Exp Ther< MedlineTA>J Pharmacol Exp Ther, 2003
- Based on a rational design where the extracellular domains of dimeric erythropoietin  [ **EPO** ]  receptor  **EpoR**  or gp130 were substituted with heterodimeric V H V L regions of anti hen egg lysozyme HEL antibody and  **EpoR**  D2 domains, the genes encoding the chimeras as well as a model transgene, enhanced green fluorescent protein EGFP, were retrovirally infected into IL 3 dependent Ba F3 cells followed by direct HEL selection in the absence of IL 3.  Ref: 12655020 Nucleic Acids Res, 2003
- Our results suggest that increased expression of  **Epo**  and  **EpoR**  may play a significant role in cervical carcinogenesis and tumor progression.  Ref: 12759237 Am J Pathol, 2003
- METHODS AND RESULTS Specimens of primary gastric adenocarcinomas obtained from 40 patients who had undergone curative gastrectomy were investigated immunohistochemically by using anti CD31 and anti  **Epo**  R  [ **EPOR** ]  antibodies.  Ref: 12605644 Histopathology, 2003
- In addition, adhesion of 32D  **EpoR**  Wt cells to immobilized H 296, a recombinant fibronectin peptide specific for alpha4beta1, induced tyrosine phosphorylation of Jak2, the erythropoietin  [ **EPO** ]  receptor  **EpoR** , and the IL 3 receptor beta subunit as well as Pyk2, Shc, and Cbl.  Ref: 12589802 Biochem Biophys Res Commun, 2003
- Acting via its receptor  **EpoR** ,  **Epo**  up regulates bcl 2 and inhibits apoptosis of erythroid cells and rescues neurons from hypoxic damage.  Ref: 12759237 Am J Pathol, 2003
- The authors examined pancreatic islets for the presence of  **EPO**  R  [ **EPOR** ]  to determine whether these cells are protected by  **EPO**  from cytokine induced apoptosis.  Ref: 12717230 Transplantation, 2003
- The proliferation and survival of hematopoietic cells is strictly regulated by cytokine growth factors that act through receptors of the Type I cytokine receptor family, including erythropoietin  [ **EPO** ]   **Epo**  and its receptor,  **EpoR** .  Ref: 12746455 J Biol Chem< MedlineTA>J Biol Chem, 2003
- **Epo**  s elimination kinetics was studied using a very sensitive tracer interaction method TIM before and after chemical ablation of BM as an indirect way of evaluating the  **EpoR**  through an assortment of PK parameters VM, KM, K, Cl used in differentiating the  **EpoR**  population in newborn and adult sheep.  Ref: 12750427 J Pharmacol Exp Ther< MedlineTA>J Pharmacol Exp Ther, 2003
